# Serotonin 1B Receptor Gene (*HTR1B*) Methylation as a Risk Factor for Callous-Unemotional Traits in Antisocial Boys

**DOI:** 10.1371/journal.pone.0126903

**Published:** 2015-05-18

**Authors:** Caroline Moul, Carol Dobson-Stone, John Brennan, David J. Hawes, Mark R. Dadds

**Affiliations:** 1 School of Psychology, University of New South Wales, Sydney, Australia; 2 Neuroscience Research Australia, Sydney, Australia; 3 School of Medical Sciences, University of New South Wales, Sydney, Australia; 4 School of Psychology, University of Sydney, Sydney, Australia; Georgia State University, UNITED STATES

## Abstract

The serotonin system is thought to play a role in the aetiology of callous-unemotional (CU) traits in children. Previous research identified a functional single nucleotide polymorphism (SNP) from the promoter region of the serotonin 1B receptor gene as being associated with CU traits in boys with antisocial behaviour problems. This research tested the hypothesis that CU traits are associated with reduced methylation of the promoter region of the serotonin 1B receptor gene due to the influence of methylation on gene expression. Participants (*N* = 117) were boys with antisocial behaviour problems aged 3-16 years referred to University of New South Wales Child Behaviour Research Clinics. Participants volunteered a saliva sample from which the genotype of a SNP from the promoter region of the serotonin 1B receptor gene and the methylation levels of 30 CpG sites from 3 CpG regions surrounding the location of this polymorphism were assayed. Lower levels of serotonin 1B receptor gene methylation were associated with higher levels of CU traits. This relationship, however, was found to be moderated by genotype and carried exclusively by two CpG sites for which levels of methylation were negatively associated with overall methylation levels in this region of the gene. Results provide support to the emerging literature that argues for a genetically-driven system-wide alteration in serotonin function in the aetiology of CU traits. Furthermore, the results suggest that there may be two pathways to CU traits that involve methylation of the serotonin 1B receptor gene; one that is driven by a genotypic risk and another that is associated with risk for generally increased levels of methylation. Future research that aims to replicate and further investigate these results is required.

## Introduction

Psychopathy is commonly defined as comprising two factors. Factor one concerns features of personality including a lack of empathy and a cold, manipulative interpersonal style. Factor 2 includes antisocial behaviour, impulsivity, and a history of criminal behaviour. Research has demonstrated that the personality traits associated with psychopathy are themselves a risk factor for chronic and serious antisocial behaviour problems [1, 2]. The developmental analogue of psychopathic personality traits are described as callous-unemotional (CU) traits and, like their adult counterparts, include limited empathy, a lack of guilt and remorse and a cold interpersonal style. The antisocial behaviours demonstrated by children with high levels of CU traits have been shown to have a high genetic loading [3] and to be less responsive to treatments [4]. As such, the most recent edition of the diagnostic and statistical manual for mental disorders (DSM-5) has, for the first time, included callous-unemotional traits as a specifier to conduct disorder [5].

It is commonly accepted that genetics play a substantial role in the aetiology of psychopathy, and CU traits in particular [3, 6, 7]. One neurochemical system that has been implicated both theoretically [8, 9, 10] and empirically [11, 12, 13, 14] in CU traits and psychopathy is the serotonin system. For example, recent research identified an association between SNPs in the genes encoding serotonin receptor 2A (*HTR2A*) and serotonin receptor 1B (*HTR1B*) and CU traits in children with antisocial behaviour problems [12].

*HTR1B* is of particular interest because it has been found to be linked with behaviours and characteristics commonly associated with CU traits. Most notable are the animal studies which have demonstrated that mice without the serotonin 1B receptor gene show significantly elevated levels of aggression and lower levels of anxiety [15, 16]; both of which are typically associated with psychopathy. Studies in humans have also found an association between *HTR1B* and impulsive aggression [17, 18, 19] suggesting that serotonin 1B receptors may be involved in the control of aggression and impulsivity in humans. Given that the *Htr1b* knock-out mice showed elevated aggression, it is fair to assume that the association between gene expression and phenotype would be equivalent in humans—reduced expression of *HTR1B* associated with higher levels of aggression and impulsivity. More specifically, SNP rs11568817 itself has been associated with traits linked with psychopathy including; alcohol dependence [20], self-reported anger and hostility in young men [18] and autism spectrum disorder [21] which is characterised by deficits in empathy that overlap those found in people with high CU traits.

The *HTR1B* SNP rs11568817 is of specific interest as it is known to have a functional role in the serotonin system. The minor allele creates a transcription factor binding site that results in a 2.3 fold increase in gene expression [22]. Thus, the results from previous research [12] would suggest that *increased* expression of *HTR1B* that is associated with the minor allele of rs11568817 could be a causal factor in the aetiology of callous-unemotional traits. This is in opposition to previous research to suggest that *decreased* expression of *HTR1B* is associated with increased aggression and impulsivity—both of which are behavioural features of psychopathy. There is, however, some animal research to support this opposing hypothesis. It has been demonstrated that *increased* expression of *HTR1B* in rats decreases fear-potentiated startle [23], a response that is also reliably reduced in people with high levels of CU traits [24]. It is worth remembering here that CU traits are thought to be the developmental analogue of psychopathic personality traits in adults. As described previously, psychopathy comprises two factors; the first of which concerns features of personality and interpersonal style (CU traits) and the second describes behaviours such as impulsivity and antisocial acts. This distinction between features of personality and those of antisocial behaviour allows for both over- and under-expression of *HTR1B* to be associated with the first and second factor of psychopathy accordingly.

If rs11568817 is associated with CU traits due to a functional effect on the expression of *HTR1B* then it is possible that methylation of the gene may also be associated with CU traits. Methylation (the addition of methyl groups to the DNA molecule) is a process that is typically, although not without exception, associated with reduced gene expression via lessened gene transcription; the more methylation the less the gene is expressed. In simple terms, in order for a gene on the DNA molecule to be expressed (e.g. to produce the protein the gene codes for) the DNA must first be transcribed. Methyl groups bind to the DNA molecule at CpG dinucleotides and limit the extent to which the DNA is transcribed. Once thought to be a static process occurring just once during embryogenesis, methylation is now recognised to have a dynamic component responding differentially to environmental variables throughout development. As Szyf [25] describes, “…DNA methylation might be diversifying genome function not only in response to innate developmental programs but also in response to environmental exposures enabling the same genome to express different phenotypes.” As such, DNA methylation is an epigenetic process influenced by both the genetic code of the individual and by the internal and external environment to which they are exposed. It is thought that the development of CU traits, and of psychopathic personality traits in adults, may be differentially influenced by environmental factors such as childhood maltreatment and adversity depending on the genetic profile of the individual—some individuals being at risk of developing high CU traits as a result of heritable genetic factors and other individuals being at risk when exposed to adverse early experiences. As such, epigenetic processes such as DNA methylation are prime candidates for the aetiological mechanisms driving the development of CU traits. Indeed, childhood adversity has been associated with hypermethylation and the reduced expression of *HTR1B* [26, 27] and recent research has identified methylation of the promoter region of the oxytocin receptor gene as being associated with the development of CU traits in children [28, 29].

Previous research conducted by this group [12] demonstrated that boys with high levels of CU traits were significantly more likely to be heterozygous for rs11568817 than homozygous. Given the rarity of true heterozygous effects and the small sample size, this previous result suggests that it is the presence of the minor allele that is a risk factor for CU traits. As it has been shown that the rs11568817 minor allele (G) leads to an increase in gene expression [22] we tested the hypothesis that increased expression of *HTR1B* is associated with higher levels of CU traits. As promoter methylation generally diminishes gene expression [25], this hypothesis would equate to lower levels of *HTR1B* methylation being associated with higher levels of CU traits.

## Methods and Materials

### Ethics Statement

Ethics approval was from the Human Research Ethics Committee of the University of New South Wales (UNSW). Primary caregivers provided written informed consent to take part in the research and also provided written informed consent on behalf of their participating child. Adolescents (over the age of 12) were required to provide independent written informed consent. The donation of a saliva sample was voluntary and participants and their parents were informed that choosing not to donate a biological sample would have no impact on their relationship with UNSW or their inclusion in the research program.

### Participants

All participants were referred to UNSW Child Behaviour Research Clinic (CBRC) or Royal Far West Children’s Hospital (RFW), Sydney. Participants for this study comprised (*N* = 48) boys from the original investigation of the association between CU traits and serotonin system SNPs [12] who were not taking selective serotonin reuptake inhibitors (SSRI) and for whom methylation data was available, in addition to 69 boys whose genetic data was collected after the publication of the original investigation and for whom both SNP and methylation data were available (methylation analyses were not able to be run on samples for which there was insufficient DNA remaining after SNP analyses had been completed). As a result, the total sample size for this research was *N* = 117. Inclusion criteria were as follows: 1) referral for externalizing behaviour problems; 2) aged from 3–16 years (*M* = 8.42, *SD* = 3.05); 3) no major neurological/physical illness; 4) all known (at least 3) grandparents of Caucasian background; 6) no use of SSRI or antipsychotic medication. Boys taking other prescription medications were included (*N* = 48) as their exclusion may have resulted in a biased sample. Boys had a primary diagnosis of ODD/CD (*N* = 57), ADHD (*N* = 33), Anxiety or Depression (*N* = 11), Autism Spectrum Disorder (*N* = 15). Comorbidity in this sample was high, 81 of the boys had two or more diagnoses. Diagnostic and descriptive data for the sample is displayed in Table 1.

**Table 1:** Mean Diagnostic and Environmental Variables for the Sample (N = 117).

	ODD/CD	ADHD	ASD	Anx/Dep	CU	QFE	ABS
**Mean**	2.94	1.89	0.83	1.13	7.42	71.76	7.28
**SD**	1.86	2.00	1.67	1.76	3.09	15.07	2.75
**Range**	0–6	0–6	0–5	0–5	0–14	10–90	1–10

Note: ODD = oppositional defiant disorder, CD = conduct disorder, ASD = autism spectrum disorder, Anx/Dep = anxiety or depression, CU = callous-unemotional traits, QFE = quality of the family environment, ABS = Australian Bureau of Statistics Index of Area.

### Measures

Parents completed the Antisocial Processes Screening Device (APSD) [30] and the Strengths and Difficulties Questionnaire [31]. These measures were chosen as they measure traits and behaviours associated with psychopathy and have been shown to be reliable in measuring these traits in child populations [32, 33, 34]. CU traits were rated by parents using the UNSW system of combining items from the APSD [30] and prosocial scale of the SDQ [31]. This method has been validated by factor analysis [32] and has been used in previous research [32, 35, 36]. The UNSW CU scale uses items from the CU subscale of the APSD and reverse-coded items from the prosocial scale of the SDQ to improve upon the internal consistency of the CU subscale of the APSD and has good stability over a 1 year period (*r* = 0.65) (see 32 for details regarding the development and psychometric properties of this CU scale). In this sample (*N* = 117) the internal consistency of the UNSW CU scale was acceptable (*α* = 0.76) and the mean, standard deviation and range of scores are shown in Table 1. Previous research suggests a range between the top 45% and 20% of clinic-referred aggressive/antisocial groups to represent high CU traits [2, 5, 32]; thus, boys with a CU traits score equal to or less than the 33^rd^ percentile were categorised as the low CU group. Boys with a CU traits score greater than this value were categorised into the high CU group.

Diagnoses were made using DSM-IV criteria by the assessing psychologist using The Diagnostic Interview Schedule for Children, Adolescents, and Parents (DISCAP) [37] with parents, and the child for those older than 8 years. Diagnoses were checked by having a second diagnostic team make an independent diagnosis. Kappa agreement across UNSW child mental health services on primary and secondary diagnoses were 0.772 and 0.770 respectively. Participants were rated by clinical psychologists, blind to levels of CU traits, on levels of antisocial behaviour problems (Oppositional Defiant Disorder (ODD) and Conduct Disorder (CD)), Attention Deficit Hyperactivity Disorder (ADHD), autism spectrum disorders (ASD) and anxiety and depression. Ratings were made on a scale of 0 to 6 with a rating of 4 or more indicating clinical levels of severity and a rating of 3 indicating borderline clinical severity.

Adversity for the child was measured using the Quality of the Family Environment (QFE) [38], a clinician rating scale of the lowest quality of family environment to which the child was exposed during a substantial period (at least 1 year) before the age of 12. Ratings were made by a second naïve clinician on a subset of cases (*r* = 0.96). The parent also provided information regarding their home address. This information was used in conjunction with the Australian Bureau of Statistics (ABS) Socio-Economic Index of Area [39] to estimate the quality of the child’s current surrounding environment. This scale provides a rating of the average standard of living for a given area on a ten-point scale with 1 indicating disadvantage and 10 indicating advantage. Sample characteristics are shown in Table 1.

### Genotyping

Participants volunteered a saliva sample for genetic analyses. Saliva samples were collected using Oragene saliva collection kits (http://www.dnagenotek.com/). DNA extraction rates were > 95%. Genotypes of rs11568817 were determined using iPLEX Gold primer extension followed by mass spectrometry analysis on the Sequenom MassARRAY system (Sequenom, San Diego, CA) by the Australian Genome Research Facility (AGRF: http://www.agrf.org.au/). Genotypes of rs11568817 were found to be in Hardy-Weinberg equilibrium (*p* = 0.64).

### Methylation

Methylation levels were quantified using the EpiTYPER assay on the Sequenom MassARRAY system at the AGRF. Methylation was assayed for 30 CpG dinucleotides within three CpG regions surrounding the location of rs11568817. Two CpG regions are from the promoter region of *HTR1B* (as described by Takai et al., [40] and Dammann et al., [41]) and the third is located in area H6, a CpG-rich region close to the promoter region for which hypermethylation is thought to be associated with loss of gene expression (see [40]). The locations of these CpG regions (with reference to rs11568817) are described in Tables 2 and 3.

**Table 2:** The Location of the CpG Regions in *HTR1B* (location of rs11568817 = GRCh37/hg19 chr6: 78173382).

**CpG region location**	**HTR1B region**	**Forward primer**	**Reverse primer**	**Reference**
chr6:78,173,039–78,173,227	Promoter	5’-GGAATTATTAATTGGGGATAAATTTG-3’	5’-CAAAAAATAAATTAACTTAAAAAACCCAAA-3’	Dammann et al. (2011)
chr6:78,173,927–78,174,170	Promoter	5’-GGAGTTTTTTTGGTTAGGAAAGG-3’	5’-CGACACACTAAAAAAAAACAAAT-3’	Takai et al. (2001)
chr6:78,176,345–78,176,539	H6	5’-ATTTGTTTGGAGGTTTTTTTTAG-3’	5’-TTCCTTTCTCACTTTAAAACACA-3’	Takai et al. (2001)

**Table 3:** The Location of the CpG Sites in *HTR1B*.

**Sequence start location**	**Region**	**Number of base pairs in sequence**	**gDNA sequence (CpG sites in bold and numbered)**
**78,173,039**	Promoter	246	GAGGATAAGTTGGCTTGAGGAACCCAGGTCT**CG** ^**1**^GAGCC**[CGCG**GG**CG**G**CG]** ^**2**^GTGGAGCGCACTGAGCACC**CG** ^**3**^GTTCCTCCATGGCTCTCCT**CG** ^**4**^CCCCAGCTC**CG** ^**5**^GAGCGCAGCTCTTGGGCATGGAGCGGACGAAGGAGAGGG**CG** ^**6**^GAAGGAC**[CG**TGG**CG]** ^7^ATCGCAGGTTTGTCCCCAGTTGATAGTTC**CG** ^**8**^TGAGTTCCTCAATTATTCCTC**CG** ^**9**^CCCAGGTTCACAGCTGAAACTAGAGGTCATGGGTG
**78,173,382**	Promoter	1	T (location of rs11568817)
**78,173,927**	Promoter	336	CACACTGGGAAGGGGCAGGTGTCTGGGGGAGCTGGAGGGACGCGGCGTGCACCAGCC**CG** ^**10**^GGAGAAG**[CG**TG**CG]** ^**11**^AGCTACGGAGCAGATGGGCTCTTTATATAGAGTCCAGGTC**[CG**GTC**CG]** ^**12**^CCAGAGCAGTGCAACTCGTGCGGCGCGGCGGGTGCGGA**[CG**CC**CG]** ^**13**^CTCCCCATCACCTTCCCTTTTTCTCTCTCCCTCCCTCTTTTCCCCCCGCCCTGTTCCTTTCCTGGCCAAGGAAGCTCC**[CG**C**CG]** ^**14**^GGTCCTAC**CG** ^**15**^CCCTATCTCCC**CG** ^**16**^CTTCCCACCCTGGGCCATCCTCCCAGCCCTGACAACCTGGAGCTGCATTTCTGGTCAGGGGAAGAAG
**78,176,345**	H6	195	ACTTGCCTGGAGGTTCCTTCTAG**[CG**C**CG]** ^**17**^CTGGGGACTGCAGAGCTGGCACTTGGACCTTACCCAGCGACCCCTACTC**CG** ^**18**^AAGGGGTGACCCAGGCACAGGCGACTTTACAGGGTCTCTTTCTCTCTTGGGTGTTTCACACACATG**CG** ^**19**^CCTCTGTGCAAACACCTATGTTTTATGTGTTTTAAAGTGAGAAAGGAA

Silent signals occurred for 2 dinucleotides, 5 other dinucleotides had masses that were either too large or too small to be assayed and another 4 CpG sites failed, leaving usable data for 19 CpG dinucleotides. Reliability of the methylation assays was checked for each CpG site. Epitect control DNA samples (Qiagen) known to be fully methylated and fully unmethylated were assessed in parallel to the study DNA to determine the upper and lower limits of detection for each assay.

#### Creating an index of methylation

In order to create a single index of *HTR1B* methylation across the three CpG regions, a principal components-based analysis was conducted using all 19 CpG sites (see [27] for previous use of this scaling method). The weighted principal component was then used to index the overall methylation of *HTR1B*. As listwise inclusion was used, only participants with methylation data for each of the 19 CpG sites were included in statistical analyses using this overall methylation index (*N* = 77). The factor loadings of each CpG site onto the principal component are displayed in Table 4.

**Table 4:** The Factor Loadings of each CpG Site onto the Principal Component of Overall *HTR1B* Methylation.

**CpG site**	**HTR1B region**	**CpG reference**	**Principal Component Coefficient**	**Mean % methylation level**
**1**	Promoter	Dammann 1	.285	1.98
**2**	Promoter	Dammann 2–5	.738	5.34
**3**	Promoter	Dammann 7	.450	3.41
**4**	Promoter	Dammann 8	.369	3.70
**5**	Promoter	Dammann 9	.381	0.90
**6**	Promoter	Dammann 13	.207	1.19
**7**	Promoter	Dammann 14/15	-.119	7.32
**8**	Promoter	Dammann 17	.098	1.38
**9**	Promoter	Dammann 18	.024	8.04
**10**	Promoter	Takai 4	.347	1.45
**11**	Promoter	Takai 5/6	.408	3.99
**12**	**Promoter**	**Takai 8/9**	-.783	11.13
**13**	Promoter	Takai 16/17	.580	1.87
**14**	**Promoter**	**Takai 19/20**	-.708	4.75
**15**	Promoter	Takai 21	.061	2.45
**16**	Promoter	Takai 22	.283	6.41
**17**	H6	Takai 1/2	.489	1.92
**18**	H6	Takai 4	.516	5.06
**19**	H6	Takai 6	.061	3.17

Note: CpG sites in bold font were found to be significantly associated with callous-unemotional traits

## Results

### Relationship of CU traits to rs11568817 genotypes

Before assessing the relationship between *HTR1B* methylation and CU traits, we first checked if the previously found association between genotypes of SNP rs11568817 and CU traits was conserved in this sample which overlapped with the original sample in which this relationship was found. Boys with high levels of CU traits (the top third of CU trait scores; *N* = 39) were significantly more likely to be heterozygous (GT) for SNP rs11568817 than participants without high levels of CU traits (the bottom and middle thirds of CU trait scores) (*χ* ^*2*^ (1, *N* = 117) = 3.44, *p* = 0.049). The effect size of the association was smaller than in the original sample [12]. This is possibly due to the purer diagnostic criteria used in the original research; the original sample comprised 133 boys all of whom had a diagnosis of either conduct disorder or oppositional defiance disorder.

As found previously, this effect was specific to heterozygotes. An additive genetic model (in which each copy of the minor allele is hypothesised to carry an increased risk for high CU traits such that minor homozygotes would carry twice the risk for high CU traits as heterozygotes) was tested using binary regression and including ADHD severity as a covariate. The additive model was non-significant (*χ*2 (3, *N* = 117) = 5.09, *p* = 0.165, Cox & Snell *R* ^2^ = 0.044) with genotype non-significant as a predictor of high CU traits (Wald = 4.069, *df* = 2, *p* = 0.131). The recessive genetic model was also tested to verify that the increased risk of high CU traits was not simply for all carriers of the minor allele (minor homozygotes and heterozygotes). This model was not significant (*χ* ^*2*^ (1, *N* = 117) = 0.16, *p* = 0.42). It should be noted here that the heterozygous effect was not found in the subsample of participants for whom an overall mean methylation index was available (χ2 (1, N = 76) = 2.79, p = 0.078). The direction of the effect was, however, in the same direction as that found in the whole sample, with more high CU participants than expected being heterozygotes.

### Covariates associated with methylation

Next, possible environmental and diagnostic covariates were assessed for their association with overall *HTR1B* methylation level by means of correlation analyses for continuous covariates and univariate analyses of variance for nominal variables. Environmental and diagnostic variables were; age, QFE, Socio-Economic Index of Area, medication usage (yes or no), CP diagnostic severity, ADHD diagnostic severity, Anxiety/Depression diagnostic severity, and ASD diagnostic severity. Of these variables only the severity of ADHD symptoms approached significance as a correlate of *HTR1B* methylation (*N* = 76, *r* = 0.21, *p =* 0.068) and, to be cautious, was used as a covariate in following analyses.

### The association between genotype and methylation

**Fig 1:**
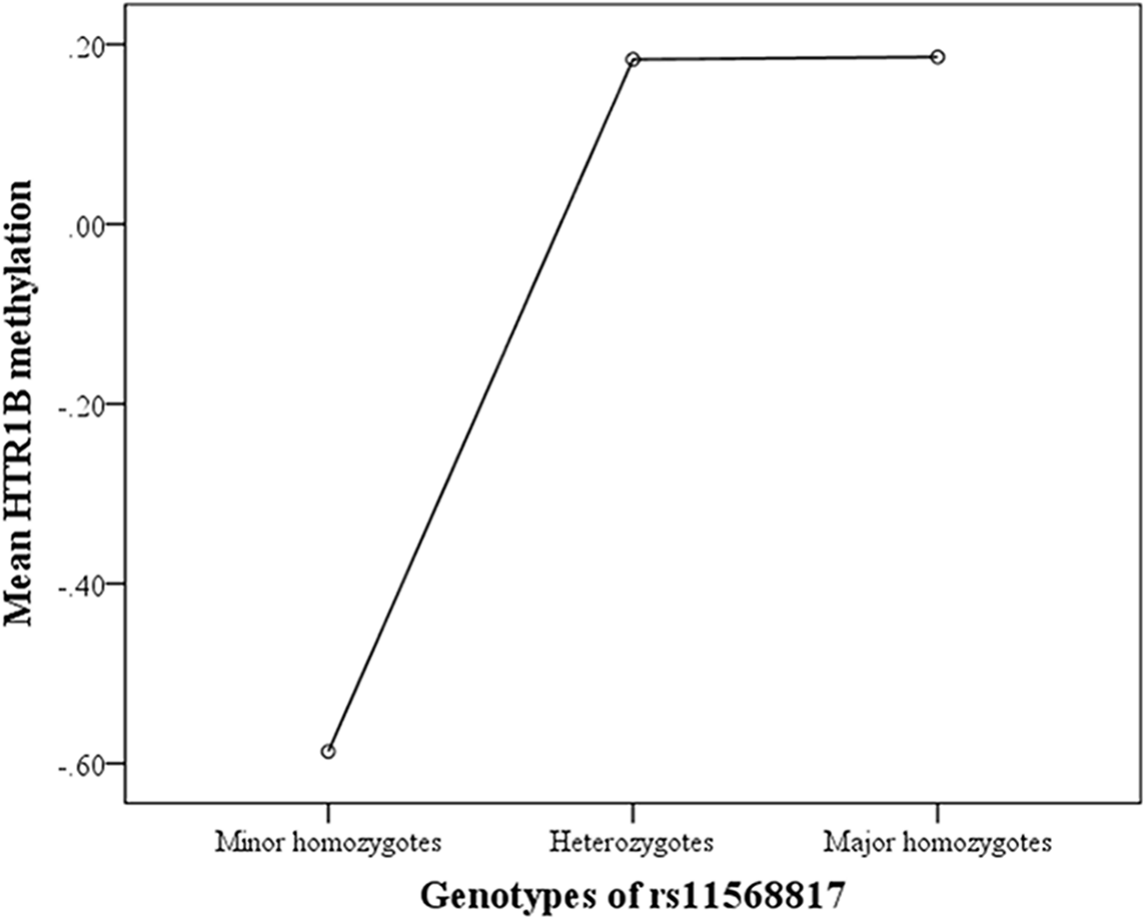
Mean Methylation Levels of *HTR1B* According to Genotype. Mean *HTR1B* methylation is represented by factor scores. Minor homozygotes (*N* = 17), heterozygotes (*N* = 31), major homozygotes (*N* = 28).

In order to determine whether there was a genotypic effect on *HTR1B* methylation an analysis of variance was conducted, with genotypes of rs11568817 as the independent factor and *HTR1B* methylation as the dependent variable. Genotype was found to be a significant predictor of methylation level (*F*(2,73) = 4.26, *p* = 0.01). Follow-up pairwise comparisons showed that minor homozygotes (*N* = 17, *M* = -0.586, *SD* = 1.13) had significantly lower mean levels of methylation than either heterozygotes (*N* = 31, *M* = 0.183, *SD* = 1.00) (Mean difference = -0.770, *p* < 0.01, 95% CI (-1.35 to -0.19)) or major homozygotes (*N* = 28, *M* = 0.186, *SD* = 0.797) (Mean difference = -0.772, *p* = 0.01, 95% CI (-1.36 to -0.18)). There was no significant difference in mean levels of methylation between heterozygotes and major homozygotes (*p* = 0.99). Fig 1 displays the mean methylation levels for genotypes of rs11568817.

### The association between *HTR1B* methylation and callous-unemotional traits

To test whether methylation of *HTR1B* was a significant predictor of CU traits, an analysis of variance was conducted with CU traits as the dependent variable, ADHD severity included as a covariate, and genotype, *HTR1B* methylation and an *HTR1B* methylation by genotype interaction variable entered as independent predictors. This interaction term was included because of the previously found association between genotype and methylation (homozygotes had lower levels of methylation than either heterozygotes or major homozygotes).

No significant main effect was found for either the genotype (*F*(2,69) = 0.296, *p* = 0.744) or for *HTR1B* methylation (*F*(1,69) = 0.529, *p* = 0.469). ADHD severity was non-significant as a covariate (*F*(1,69) = 0.152, p = 0.697). The genotype by methylation interaction term was, however, found to be a significant predictor of CU traits (*F*(2,69) = 7.477, *p* = 0.001, *η*_*p*_^*2*^ = 0.178, observed power = 0.933).

**Fig 2:**
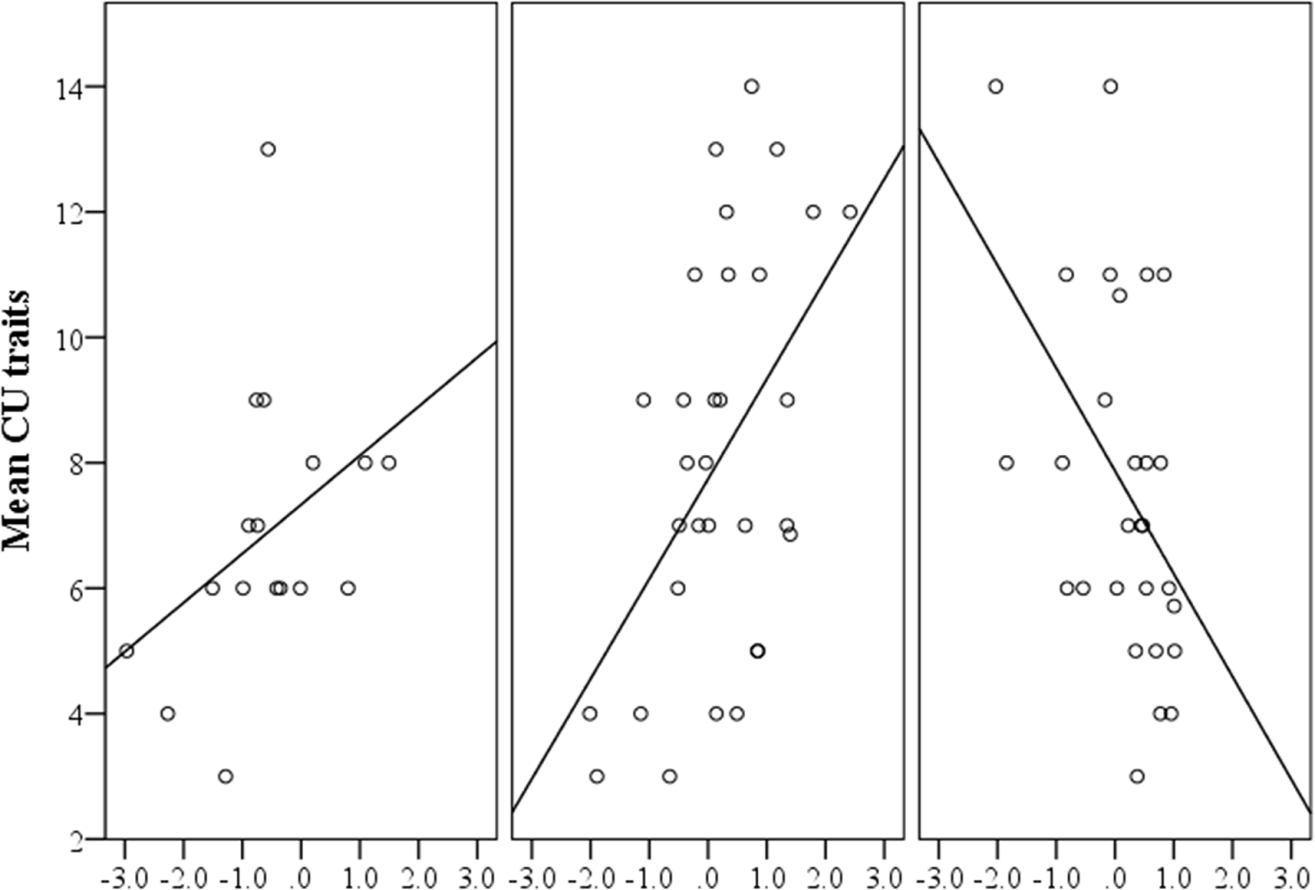
The Relationship between Mean CU Traits and *HTR1B* Methylation According to Genotype. CU = callous unemotional, x-axis shows mean methylation factor scores for each of the genotypes of rs11568817. Left panel = minor homozygotes (*N* = 17), middle panel = heterozygotes (*N* = 31), right panel = major homozygotes (*N* = 28).

In order to investigate the nature of the interaction effect, the correlation coefficients for the relationship between CU traits and *HTR1B* methylation were determined independently for each genotype. CU traits were found to be significantly positively correlated with *HTR1B* methylation only for heterozygotes of rs11568817 (*N* = 31, *r* = 0.495, *p* = 0.005). The relationship between CU traits and *HTR1B* methylation for minor homozygotes was found to be positive however there was insufficient power (*N* = 17) to interpret the significance of the correlation. Major homozygotes displayed a significant negative correlation (*N* = 28, *r* = -0.423, *p* = 0.025). The results are displayed in Fig 2.

### Post-hoc analyses of individual CpG sites

We were interested to know whether the methylation of certain CpG sites was driving the association between CU traits and overall *HTR1B* methylation. To test the CpG x genotype interaction on CU traits, individual analyses of variance were conducted for each of the 19 CpG sites. In each analysis of variance CU traits was included as the dependent variable, ADHD severity was included as a covariate, and genotype, CpG site methylation and a CpG site methylation by genotype interaction variable were entered as independent predictors. Bonferroni corrections for multiple comparisons (*N* = 19) were applied (significant *p* < 0.0026). Of all 19 CpG sites, methylation of only 4 sites interacted with the genotype to be significant predictors of CU traits at *p* < 0.05. These sites were; 10, 12, 13 and 14 (see Table 3 for description of these sites). Of these four CpG sites, only the interaction effect of 2 sites (12 and 14) survived correction for multiple comparisons. For CpG site 12, *F*(2,75) = 6.597, *p* = 0.002, *η*_*p*_^*2*^ = 0.150, observed power = 0.900, and for site 14, *F*(2,75) = 11.403, *p* < 0.001, *η*_*p*_^*2*^ = 0.233, observed power = 0.991.

**Fig 3:**
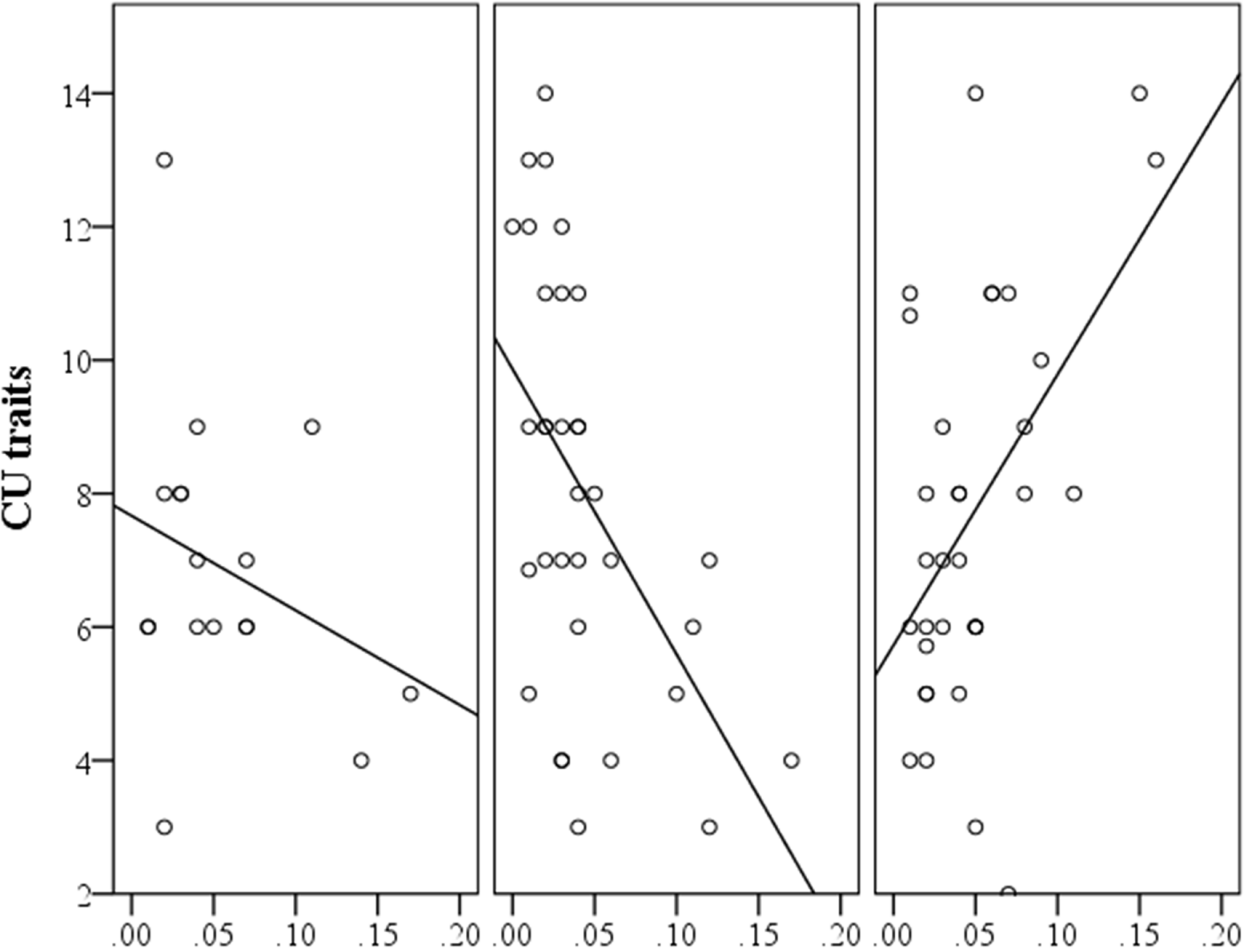
The Relationship between CU Traits and Methylation of CpG Site 12 According to Genotype. CU = callous unemotional, x-axis shows methylation levels of site 12 for each of the genotypes of rs11568817. Left panel = minor homozygotes, middle panel = heterozygotes, right panel = major homozygotes. Correlation coefficients are significant for heterozygotes (*N* = 33, *r* = -0.529, *p* = 0.002) and major homozygotes (*N* = 28, *r* = 0.497, *p* = 0.003).

It was noted that these two CpG sites (12 and 14) both loaded negatively onto the principal component of overall *HTR1B* methylation (see Table 3). As such, the moderating effect of genotype on the relationship between methylation levels at these sites and CU traits (shown in Fig 3) is in the opposite direction to that described using an overall principal component index of methylation.

Further post-hoc analyses of variance were used to determine whether the genotypic effect that was found for overall *HTR1B* methylation was inverted for these two CpG sites. There was no significant genotypic effect on methylation levels at CpG 14 but there was a significant effect of genotype on methylation levels at CpG 12, (*F*(2,80) = 4.21, *p* = 0.018). Minor homozygotes had significantly higher mean levels of methylation (*N* = 17, *M* = 0.158, *SD* = 0.11) than either heterozygotes (*N* = 33, *M* = 0.099, *SD* = 0.07) or major homozygotes (*N* = 33, *M* = 0.097, *SD* = 0.06). As expected from the factor loadings of this site, this is the inverse pattern of results to that found for overall *HTR1B* methylation.

## Discussion

We hypothesised that methylation of *HTR1B* would be inversely associated with CU traits. This was predicted as previous research has identified a putative heterozygous effect of SNP (rs11568817) in the promoter region of *HTR1B* [12], the minor allele of which leads to increased *HTR1B* expression. Thus it was proposed that CU traits were associated with greater expression of *HTR1B* via less methylation of the promoter region of this gene. This hypothesis was supported but the relationship was found to be moderated by the genotype of rs11568817.

With regards to overall mean methylation, our results supported previous findings [17, 42, 19] to suggest that less *HTR1B* expression is associated with risk for CU traits. For minor homozygotes and heterozygotes, overall *HTR1B* promoter methylation was positively associated with CU traits. Only major homozygotes demonstrated the inverse relationship between overall methylation and CU traits that we predicted. The results were further complicated by the finding that the association between methylation and CU traits was carried principally by two CpG sites for which levels of methylation were negatively associated with overall *HTR1B* methylation. Specifically, methylation of CpG sites 12 and 14 was inversely associated with CU traits for minor homozygotes and heterozygotes only. The genotypic effect on overall *HTR1B* methylation (minor homozygotes had significantly lower levels of overall *HTR1B* methylation than either heterozygotes or major homozygotes) was also found to be inverted for CpG site 12 with minor homozygotes having greater levels of methylation at this site than either heterozygotes or major homozygotes.

A genotype x methylation interaction effect on CU traits was found for CpG sites 12 and 14. The relationship between methylation and CU traits was negative for carriers of the minor allele (although did not reach significance for minor homozygotes) but positive for major homozygotes. Given the small sample size for minor homozygotes (*N* = 17) it is possible that the correlation for this genotype was non-significant due to insufficient power.

Given the results we suggest that it is possible that methylation of CpG sites 12 and 14 may serve to counteract the increased transcription of *HTR1B* that is created by the presence of the minor allele at rs11568817. In terms of a “risk profile” for CU traits we suggest that the presence of the minor allele (which results in an extra transcription factor binding site) serves as a risk factor for high CU traits. Further, we suggest that methylation of CpG sites 12 and 14 decreases transcription factor binding to this site and so reduces the risk for high CU traits. For major homozygotes, who do not possess the genotypic risk factor for high CU traits, methylation at CpG sites 12 and 14 serves no functional purpose and instead, given the factor loadings of these sites, represents less methylation of *HTR1B* overall. Less methylation of *HTR1B*, in general, would result in greater gene transcription hence the negative association with CU traits for major homozygotes.

Interestingly, the genotypic effect on methylation at CpG site 12 (minor homozygotes had higher levels of methylation) provides a plausible explanation for the apparent heterozygous effect of the genotype for CU traits. If the presence of the minor allele is a genetic risk factor for CU traits and the methylation of CpG site 12 reduces this risk then the greater levels of methylation at this site associated with minor homozygotes would serve to leave only heterozygotes as having the highest genetic risk profile for CU traits.

This explanation for the relationships between genotypes of rs11568817, methylation of CpG sites 12 and 14 and CU traits is theoretical and based on the hypothesis that CU traits are associated with increased expression of *HTR1B*. It is beyond the scope of this research to determine how methylation of CpG sites 12 and 14 could functionally influence the extra transcription binding site produced by the presence of the minor allele of rs11568817. It is also unclear how the methylation of CpG sites 12 and 14 is functionally associated with overall *HTR1B* methylation and what processes may lead to this differential methylation. Suffice to say that research is required at a more basic level to determine whether our proposed explanation for the results found in this research would be viable.

Previous research into phenotypes associated with the expression of *HTR1B* has identified *low* levels of *HTR1B* expression as a risk factor for aggression [17], impulsivity [42] and drug and alcohol use [19]. As each of these phenotypes is associated with psychopathy the opposing conclusion proposed by the current research—that CU traits are likely associated with greater gene expression (for carriers of the minor allele)—presents a potential problem. We would suggest that *HTR1B* expression may be differentially associated with factor 1 (personality characteristics including CU traits) and factor 2 (antisocial and impulsive behaviours) components of psychopathy. There are likely numerous independent genetic and environmental causal pathways to the development of CU traits and antisocial behaviours such that the proposed contradiction of *HTR1B* involvement in the two factors does not rule out their coexistence.

CU traits are commonly accepted as having a strong genetic basis but this does not negate the relevance of interaction effects with environmental variables such as parenting and exposure to adversity. An interesting finding of this research is that there appear to be two hypothetical routes to high levels of CU traits via the *HTR1B* gene. The first route would involve having the minor risk allele and low levels of methylation at CpG sites 12 and 14 (high levels of promoter methylation overall), the second would not include the risk allele but would be associated with high levels of methylation at CpG sites 12 and 14 (low levels of promoter methylation overall). It is unclear what processes may lead to different levels of methylation at these two sites but it is possible that stressors such as abuse and maltreatment in childhood may be an important factor. In other words, this research identifies one possible pathway that is more indicative of a genetic risk profile (akin to primary psychopathy) whereas the second pathway of high levels of methylation at sites 12 and 14 may be more associated with environmental factors and, in this respect, more representative of the proposed driving forces behind the development of secondary psychopathy. It would be beyond the scope of this research to conclude such a suggestion but the possibility that the same gene could be involved in the aetiology of high CU traits via both a genetic risk profile and by purely environmental factors is an interesting one that warrants further investigation.

This research is limited by a number of factors. The sample was restricted to Caucasian boys referred for treatment for behaviour problems. As such, it is unknown whether the results would hold true for females, for non-clinical populations and for other ethnicities. The sample was, however, heterogeneous with regards to diagnoses and symptom severity and is unlikely to be representative of any specific psychopathology. As a consequence of using a clinical sample, the number of participants was fewer than would be desired to obtain optimum power for statistical analyses. Thus, these results should be interpreted cautiously until replicated in an independent sample. However, the moderate to large effect sizes found for the genotype x CpG sites 12 and 14 interaction variables as predictors of CU traits are encouraging.

The loadings of some of the individual CpG sites onto the PCA of overall methylation were low. As such, the use of an “overall methylation” index may not be as useful as looking at methylation at individual sites. Given the results of this research it may be useful for future research and replication studies to examine only methylation at sites 12 and 14 which loaded most strongly, and negatively, onto the overall index.

This research also used methylation of DNA from saliva samples as opposed to tissue samples from discrete brain regions. Ethically, of course, saliva samples are the most appropriate form of DNA collection from child samples but methylation patterns of *HTR1B* may not be consistent across all tissue types [43]. As such, the results may represent global *HTR1B* expression but there may also be differential patterns of methylation across different tissues and within different brain regions. It is also not possible to predict the influence of altered *HTR1B* expression on serotonin neurotransmission as the function and distribution of serotonin 1B receptors is varied and multifaceted. *HTR1B* is expressed in a number of regions including the substantia nigra, the globus pallidus, the amygdala and hippocampus and at low levels in the cortex; and can have activating or inhibiting effects depending on the type of neuron on which it is expressed [43]. Research also suggests that *HTR1B* enhances serotonin transmission in the caudal parts of the brain but inhibits serotonin neurotransmission in the rostral part of the brain [44]. Suffice to say, the distribution and functional effects of *HTR1B* make it a plausible candidate as a gene involved in the aetiology of CU traits. This does not, of course, rule out other genes both within and outside of the serotonin system that were not examined in this research in the aetiology of CU traits.

This research is the first to identify an association between methylation of *HTR1B* and CU traits and supplements growing evidence to implicate the serotonin system in the aetiologies of psychopathic personality and CU traits. Further research is required to replicate these results, to determine the mechanisms that drive the association and to identify the neural systems and brain regions that are involved.
